# Association between *IL1* gene polymorphism and human African trypanosomiasis in populations of sleeping sickness foci of southern Cameroon

**DOI:** 10.1371/journal.pntd.0007283

**Published:** 2019-03-25

**Authors:** Elvis Ofon, Harry Noyes, Vincent Ebo’o Eyanga, Flobert Njiokou, Mathurin Koffi, Pythagore Fogue, Christiane Hertz-Fowler, Annette MacLeod, Enock Matovu, Gustave Simo, for the TrypanoGEN Research Group, as members of The H3Africa Consortium

**Affiliations:** 1 Molecular Parasitology and Entomology Unit, Department of Biochemistry, Faculty of Science, University of Dschang, Dschang, Cameroon; 2 Centre for Genomic Research, University of Liverpool, Liverpool, United Kingdom; 3 MINSANTE, Divisional Centre for Diseases, PNLTHA, Ministry of Public Health, Yaoundé, Cameroon; 4 Laboratory of Molecular Biology, Department of Animal Biology, Faculty of Science, University of Yaoundé I, Yaoundé, Cameroon; 5 Université Jean Lorougnon Guédé (UJLoG), UFR Environnement-Santé, Laboratoire des Interactions Hôte- Microorganismes-Environnement et Evolution (LIHME) Daloa, Côte d’Ivoire; 6 Wellcome Centre for Molecular Parasitology, University Place, Glasgow, United Kingdom; 7 College of Veterinary Medicine, Animal Resources and Bio-security, Makerere University, Kampala, Uganda; Universiteit Antwerpen, BELGIUM

## Abstract

**Background:**

Human African Trypanosomiasis (HAT) is a neglected tropical disease caused by infections due to *Trypanosoma brucei* subspecies. In addition to the well-established environmental and behavioural risks of becoming infected, there is evidence for a genetic component to the response to trypanosome infection. We undertook a candidate gene case-control study to investigate genetic associations further.

**Methodology:**

We genotyped one polymorphism in each of seven genes (*IL1A*, *IL1RN*, *IL4RN*, *IL6*, *HP*, *HPR*, and *HLA-G*) in 73 cases and 250 controls collected from 19 ethno-linguistic subgroups stratified into three major ethno-linguistic groups, 2 pooled ethno-linguistic groups and 11 ethno-linguistic subgroups from three Cameroonian HAT foci. The seven polymorphic loci tested consisted of three SNPs, three variable numbers of tandem repeat (VNTR) and one INDEL.

**Results:**

We found that the genotype (TT) and minor allele (T) of *IL1A* gene as well as the genotype 1A3A of *IL1RN* were associated with an increased risk of getting *Trypanosoma brucei gambiense* and develop HAT when all data were analysed together and also when stratified by the three major ethno-linguistic groups, 2 pooled ethno-linguistic subgroups and 11 ethno-linguistic subgroups.

**Conclusion:**

This study revealed that one SNP rs1800794 of *IL1A* and one VNTR rs2234663 of *IL1RN* were associated with the increased risk to be infected by *Trypanosoma brucei gambiense* and develop sleeping sickness in southern Cameroon. The minor allele T and the genotype TT of SNP rs1800794 in *IL1A* as well as the genotype 1A3A of *IL1RN* rs2234663 VNTR seem to increase the risk of getting *Trypanosoma brucei gambiense* infections and develop sleeping sickness in southern Cameroon.

## Author summary

Human African Trypanosomiasis (HAT), or sleeping sickness, is a parasitic disease caused by flagellated parasites of the genus *Trypanosoma*. This disease has been included into the WHO roadmap for neglected tropical diseases with elimination as a public health problem targeted for 2020 and the interruption of transmission to humans for 2030. To achieve these elimination and interruption goals, it is important to identify and understand the factors that may hamper these goals. Understanding the contribution of human genetics to the response of trypanosome infections is important for the development of new control strategies. In this study, polymorphism in seven genes was investigated between controls and sleeping sickness patients of three sleeping sickness foci of Southern Cameroon in order to see if there is any association with the development of disease. Results of this study have shown that the genotype (TT) and minor allele (T) of *IL1A* gene and the genotype 1A3A VNTR of *IL1RN* are associated with an increased risk of getting *T*. *b*. *gambiense* infections and develop sleeping sickness in major ethno-linguistic groups of the Cameroonian population. They suggest that the association between host genetic determinants and the susceptibility to *T*. *b*. *gambiense* infections could vary according to the population studied. These results will improve our knowledge on the role of human genetics determinants and the risk to be infected by *T*. *b*. *gambiense* and develop sleeping sickness. They could thus lead to the identification of novel biomarkers which could open a frame work for the development of new diagnostics, treatments and intervention strategies.

## Introduction

Human African Trypanosomiasis (HAT), or sleeping sickness, is a parasitic infection caused by flagellated parasites of the genus *Trypanosoma*. The parasites belong to *Trypanosoma brucei* complex which is subdivided into three subspecies: *Trypanosoma brucei gambiense* (Dutton, 1902) is responsible for the chronic form of the disease in West and Central Africa, *T*. *b*. *rhodesiense* (Stephen and Fantham, 1910) causes the acute form of HAT in East and South Africa whilst *T*. *b*. *brucei* is only infective to animals. These trypanosomes are transmitted through the bites of haematophagous flies of the genus *Glossina* commonly known as tsetse flies [1, 2]. HAT was considered to be under control during the 1960s, but the disease has re-emerged in the last decades as a public health problem in many sub-Saharan African countries due to the abandonment of control measures after independence and also to socio-political and environmental upheavals [2]. HAT is often fatal unless treated and is endemic in 36 sub-Saharan Africa countries with about 65 million people in more than 250 foci exposed to the risks of infections [3]. Currently, more than 98% of the reported cases are due to *T*. *b*. *gambiense* infection [3]. Control efforts undertaken by the national sleeping sickness control programs have succeeded in considerably reducing the number of cases, and less than 2200 new cases were officially reported in 2016 [4]. Sleeping sickness has been included in the WHO roadmap for neglected tropical diseases with elimination as a public health problem targeted for 2020, and the interruption of transmission to humans for 2030.

To achieve the elimination and interruption goals, it is important to identify and gain a better understanding of the clinical evolution of the disease and the factors that may hamper this goal. Addressing the contribution of human genetics to the response to *T*. *b*. *gambiense* infections is important for the development of new control strategies because a range of clinical presentations of HAT including asymptomatic carriers and spontaneous cure without treatment have been reported in West Africa [5]. Understanding the genetic bases of these new disease profiles may help to identify more susceptible populations for more effective control operation. Previous studies have identified polymorphisms in *APOL1*, *IL6*, *HLA-G* and *HP/HPR* that regulate the human susceptibility to trypanosome infections [6, 7, 8, 9, 10, 11, 12, 13]. Most of these genes seem to play important roles during *T*. *b*. *gambiense* infection [14]. For instance, HLA-G has been reported to be involved in HAT progression. In addition, *IL1* participates in macrophage activation during early *T*. *b*. *gambiense* infection in mice [15]. It also plays a key role in the recruitment of leukocytes into the CNS during *T*. *b*. *gambiense* infections [16, 17]. However, contrasting results on the association between gene polymorphisms and the risk to be infected by *T*. *b*. *gambiense* and develop HAT have been reported between countries and therefore, efforts are needed to better understand the genetic bases of human susceptibility to *T*. *b*. *gambiense* infections. In this current study, polymorphisms in seven genes were genotyped to identify any association with HAT. Our data suggest that the association between host genetic determinants and the susceptibility to be infected by *T*. *b*. *gambiense* and develop sleeping sickness could vary according to the population studied.

## Materials and methods

### Study areas

This study was conducted in three active sleeping sickness foci in the forest region of Southern Cameroon. The three HAT foci were Bipindi and Campo in the Southern region and Fontem in the South-west region of Cameroon.

The Campo focus (2°82'00"N, 9°85'20"E) is located in the equatorial forest and extends from the Atlantic coast along the Ntem river which delimits the Cameroon–Equatorial Guinea border. It is a hypo-endemic focus where no epidemic outbreak has been observed for many decades [18]. It is a cosmopolitan area with several ethnic groups (mainly the Iyassa, Kwasse, Maabi, Mvae and Ngoumba) with most of them speaking Bantu family languages. Other minor ethnic groups are semi Bantus, Sao-Sudanese and Baka [19].

The Bipindi HAT focus (3°82'00"N, 10°82'20"E) is located at about 75 km from the Atlantic coast in the South of Cameroon. Bipindi has been known as a HAT focus since 1920. During the last two decades, it was among the most active HAT foci of Cameroon with about 83 HAT cases diagnosed from 1999 to 2011 [19]. About 95% of the inhabitants of the Bipindi HAT focus are Bantu speakers and the majority belong to the Ngoumba, Nti and Fan. The remaining 5% of inhabitants are Baka, semi Bantus and Sao-Sudanese speakers.

The Fontem focus (5°40’00”N, 9°55’00”E) is located in the South-West Region of Cameroon where HAT has been known to occur since 1949 [20]. It was previously among the most active HAT foci of Cameroon [21], but in recent decades, it has become hypo-endemic with about 8 patients detected among 16,000 persons examined between 1998 and 2007 [22]. In this focus, the Mundani and Banyangi are the major ethnic groups. Other minor ethnic groups such as Bangwa and Bamileke are also found.

### Study population

The Cameroonian population is made up of more than 250 ethno-linguistic subgroups from three major ethnic groups: Bantu (e.g.: Bulu, Bassa, Bakundu, Maka, Douala), Semi Bantu (e.g.: Bamileke, Gbaya, Bamoun, Tikar) and Sudano-Sao (e.g.: Fulbe, Mafa, Toupouri, Shoa-Arabs, Moundang, Massa, Mousgoum) [11] (S1 Table). Beside these three groups, some minor groups exist such as the Baka who generally speak the Bantu languages but who are not closely related to any of these three major groups [23].The ethnic composition varies considerably between regions, HAT foci and even within the same HAT focus.

### Ethical consideration

The protocol of this study was approved by the Ethical Committee of the Ministry of Public Health of Cameroon on 21 November 2013 with a reference number N°2013/11/364/L/CNERSH/SP. The local administrative and traditional authorities of each HAT focus were also informed and gave their approval. Subsequently, the review board of the Laboratory of Microbiology and Anti-microbial Substances (LAMAS) of the Department of Biochemistry of the Faculty of Science of the University of Dschang gave its approval. All adult subjects provided informed consent, and a parent or guardian of any child participant below 18 years old provided informed consent on their behalf. Each informed consent was written because all individuals enrolled in this study gave their approval by signing an informed consent form and a Certificate of Confidentiality. In addition, an assent form was also signed by children below 18 years old. During analyses, data for each subject were anonymized.

### Sample collection

Blood samples were collected during medical surveys performed jointly with the National Sleeping Sickness Control Program of Cameroon and the research team of the molecular parasitology and entomology unit of the University of Dschang. The sampling was performed in Campo in 2014 and 2017, in Bipindi in 2015 and 2017, and in Fontem in 2015. During these surveys, all participants at risk were tested using the Card Agglutination Test for Trypanosomiasis (CATT). It was performed on blood collected by finger prick [24]. This immunological test was carried out to screen people who have been in contact with *T*. *b*. *gambiense*. It was initially performed on whole blood as described by Magnus *et al*. [24]. For all participants with a positive CATT on whole blood, blood sample was collected in EDTA tubes and a two-fold plasma dilution series in CATT buffer was tested to assess the end titer, i.e. the highest dilution still positive on plasma (CATT-P). All individuals with CATT dilution on plasma≥1/8 underwent parasitological examinations by direct examination using the capillary tube centrifugation (CTC) [25] and mini-anion exchange centrifugation technique (mAECT) [26]. Beside the CTC and mAECT, lymph node aspiration followed by a microscopic examination was performed to search for trypanosomes in all individuals showing enlarged lymph nodes. All controls and participants with a CATT dilution ≥1/8 and negative for parasitological tests were subjected to the trypanolysis test in order to confirm their status [27].

### Trypanolysis test

Ninety micro-liters of plasma sample from each control and each individual with CATT dilution ≥1/8 and negative for parasitological tests were spotted on a Whatman paper disc (divided in three equal parts with each bearing a spot of 30μl) that was sent to the “Centre International de Recherche-Développement sur l’Élevage en Zones Sub-humides (CIRDES, Bobo-Dioulasso, Burkina Faso)”. Each plasma sample was tested by the immune trypanolysis test as described by Jamonneau *et al*. [27]. It is the highly specific test for *T*. *b*. *gambiense* and constitutes a routine test for the surveillance of HAT. This test was performed on plasma as previously described by Van Meirvenne *et al*. [28] with LiTat 1.3, 1.5, and 1.6 variable antigen types (VAT).

During medical surveys, each new HAT case was defined as an individual in whom trypanosomes were seen by at least one parasitological method. Old HAT cases were also sampled. They were residents in whom trypanosomes had been previously seen by at least one parasitological test after passive or active case detection. Old HAT cases were included only if the information regarding the clinical status, the CATT and all parasitological tests were available in hospital records and in the National control program register.

Each HAT case was matched to at least three controls. This matching was done by age, sex, occupation and when possible by ethno-linguistic subgroup. A control was considered as any individual who was negative for the CATT and trypanolysis tests and all parasitological tests including CTC, mAECT and lymph node examination [11]. These controls were enrolled during medical surveys.

### DNA extraction

Five millilitres of blood were centrifuged at 3500g for 3 minutes and the buffy coat was collected. Genomic DNA was extracted from the Buffy-coat with the QIAamp DNA Blood Midi/Maxi kit (Qiagen) according to the manufacturer's instructions. The DNA was eluted with 200μl of sterile water and stored at -20°C until use.

### Selection of candidate genes, SNPs, VNTR and INDEL

For this study, seven genes containing three SNPs, three VNTRs and one INDEL (Table 1) were identified and selected based on literature searches. The selected genes and loci were associated to HAT and other diseases. The selection of *HLA-G*, *HP* and *HPR* genes as well as two different cytokines genes (*IL6* and *IL1A)* was based on their previously reported association with HAT [6, 7, 9, 10]. *HPR* and haptoglobin (*HP*) are involved in the lysis of trypanosomes and the scavenging of haem during trypanosome infections [8]. The *IL1* gene has been shown to enhance immune-modulating and stimulating effects on the TLF components and inflammatory immune response activities during HAT infection [29, 30, 31]. Associations between some polymorphic variants within these genes and the outcome of HAT have also been previously outlined [6, 32, 33]. Loci on these genes were selected after literature searches as follows: the SNPs rs1800794 of *IL1A* and rs1554606 of *IL6* and the INDEL rs371194629 in the 3’ UTR of *HLA-G* gene were selected due to their previously reported association with HAT in the DRC although *IL1A* was not associated with HAT [6, 7]. The SNP rs1679370 of *HPR* gene was selected from a study on CNV of associated with HAT [9]. Other genes such as *IL1RN*, *IL4R* and *HP* were also selected not only for their association with other diseases, but especially because HAT seems to trigger inflammatory and immunological responses with biological pathways associating the selected genes [34, 35, 36]. The polymorphic locus rs2234663 within *IL1RN* was selected for its association with *H*. *Pylori* gastric infections in Brazil [36] while rs79071878-*IL4RN* within *IL4R* gene was due to its association with type II diabetes in India [37]. HP1/2 VNTR allele of the *HP* gene was selected based on its associations with malaria [36].

**Table 1:** Candidate genes and SNPs, VNTR and indel loci identified and selected for this study.

Gene	SNP	VNTR	INDEL	Localization (GRCh37)	Characteristics	Reference
*IL1A*	rs1800794	-	-	chr2:113543273	mRNA Upstream gene variant 2kb	[6]
*IL6*	rs1554606	-	-	chr7:22768707	Intron variant	[6]
*HPR*	rs1697370	-	-	chr16:35339932	Intron variant	[9]
*IL1RN*	-	rs2234663	-	chr2:113888106	Intron variant	[36]
*IL4RN*	-	rs79071878	-	chr5:132680584	Intron variant	[37]
*HP*	-	HP1/2 alleles	-	-	VNTR	[38]
*HLA-G*	-	-	rs371194629	chr6:29798581	3’ UTR variant	[7]

SNP: Single Nucleotide Polymorphism; VNTR: Variable Number Tandem Repeats; INDEL: Insertion and Deletion; UTR: Untranslated region; GRCh37: Reference human genome build version.

### Genotyping of SNPs in *IL6*, *IL1A* and *HPR* by PCR-RFLP

In this study, the SNPs in *IL6*, *IL1A* and *HPR* were investigated by PCR-RFLP where a DNA fragment of each of these genes was amplified and subsequently digested by a specific restriction enzyme. The following primer pairs were used: IL6-PF (GTCAAATGTTTAAAACTCCCACAGGTT) and IL6–PR (GCAGCCAGAGAGGGAAAAGG) for *IL6* [6], IL1-P-PF (GGCCACAGGAATTATAAAAGCTGAGA) and IL1-P-PR (GGGAGAAAGGAAGGCATGGATTTT) for *IL1A* [6] and Hpr-F (GAGCCACAAATTCTGACGAG) and Hpr-R (TTGAGGTTCTTGAGGGCATT) for HPR. The primers Hpr-F and Hpr-R for HPR were designed with the Primer3 vs 4.1 software [39, 40].

For each of these three genes, the amplification reactions were performed in a final volume of 25 μl containing 1X of PCR buffer, 1.5 mM MgCl_2_, 20 pmol of each primer, 0.5 units of Taq DNA polymerase (Qiagen) and 5–10 ng of genomic DNA. The amplification program contained a denaturing step at 95°C for 5 min followed by 35 amplification cycles of 95°C for 45 s, 63°C (*IL6* and *IL1A*) or 60°C (HPR) for 60 s and 72°C for 60 s. A final extension step was performed at 72°C for 5 min.

PCR products were visualised by electrophoresis on 2% agarose gel containing ethidium bromide. Ten micro-litters of *IL6*, *IL1A* and *HPR* PCR products were digested with Hind III, Nco I and Bci VI respectively (all enzymes from Thermo Fisher Scientific). The digestion was done overnight at 37°C in the buffer 3.1 provided by the manufacturer. The digested products of *IL6* and *IL1A* were separated by electrophoresis on a 2% agarose gel at 100 volts for 1 h 30 min. For HPR, the digested products were resolved by electrophoresis on 3.5% agarose gel at 100 volts for 2 hours.

For rs1554606 of *IL6* and rs1800794 of *IL1A*, three different profiles were expected (Table 2): the homozygote wild type genotype with two DNA fragments of 236 and 489 bp for *IL1A*, 315 and 543 bp for *IL6*, the heterozygote genotype showing three DNA fragments of 236, 489 and 725 bp for *IL1A*, and 315, 543 and 858 bp for *IL6*, and the homozygote genotype with one DNA fragment of 725 bp for *IL1A* and 858 bp for IL6.

**Table 2:** Expected number and size of DNA fragments generated at each locus genotyped.

Markers	Gene	Locus	Size of DNA fragments in base pair			Reference
			Heterozygote genotype	Homozygote		
				wild type	mutant	
SNPs	*IL1A*	rs1800794	236/489/725	236/489	725	[6]
	*IL6*	rs1554606	315/543/858	315/543	858	[6]
	*HPR*	rs1697370	88/147/235	88/147	235	[9]
VNTR	*IL1RN*	rs2234663	410/335 410/500 410/240 410/595	410	335 500 240 595	[36]
	*IL4RN*	rs79071878	183/253	183	253	[37]
	*HP*	**-**	1757/3481	1757	3481	[38]
INDEL	*HLA-G*	rs371194629	345/359	359	345	[7]

SNP: Single Nucleotide Polymorphism; VNTR: Variable Number Tandem Repeats; INDEL: Insertion and Deletion; IL: Interleukin 6; HP: Haptoglobin; HPR: Haptoglobin related protein.

For rs1697370 of *HPR*, three different profiles were expected (Table 2): the homozygote wild type genotype with two DNA fragments of 88 and 147 bp, the heterozygote genotype showing three DNA fragments of 88, 147 and 235 bp, and the homozygote mutant genotype with one DNA fragment of 235 bp.

To minimize misinterpretation of heterozygote frequency that could result from partial digestion, the amplified product of each sample (control and HAT case) was quantified and the same amount of DNA was subjected to restriction enzyme digestion. Between different amplification and digestion series, an internal control made of sample with known genotype was added. This sample was used to control the reproducibility and digestion efficiency between different amplification and digestion series.

### Amplification of 70 bp and 86 bp tandem repeats of *IL4RN* and *IL1RN* genes

The 70 bp tandem repeat (rs79071878) region of *IL4RN* gene was amplified with IL4-70 bp-F (AGGCTGAAAGGGGGAAAGC) and IL4-70 bp-R (CTGTTCACCTCAACTGCTCC) primers [37] while the 86bp tandem repeat (rs2234663) of *IL1RN* gene was amplified with IL1RN-F (CTCAGCAACACTCCTAT) and IL1RN-R (TCCTGGTCTGCAGGTAA) primers as described by Santos *et al*. [36]. For these two genes, the PCR reactions were performed in a final volume of 25 μl contained 5–10 ng of DNA, 2.5 mM and 2 mM MgCl_2_ for *IL4* and *IL1RN* respectively, 0.2 mM of each dNTP, 20 pmol of each primer and 0.5 units of Taq polymerase (Qiagen). The amplification program was 95°C for 5 min followed by 35 cycles of 95°C for 45 s, 61°C for 45 s and 72°C for 60 s. A final extension was performed at 72°C for 5 min. PCR products were separated by electrophoresis on a 2% agarose gel at 100 volts for 1 h 30 min.

The size and number of tandem repeats were evaluated for each sample. For *IL4RN*, the PCR products of 183bp (two repeats of 70b p) and 253 bp (three repeats of 70 bp) correspond to homozygote wild type (genotype R1R1) and homozygote mutant (genotype R2R2) respectively (Table 2). Sample with two DNA fragments of 183 bp and 253 bp was considered as a heterozygote with genotype R1R2.

For *IL1RN*, different alleles with specific sizes could be generated after electrophoresis as described by Santos *et al*. [36]: alleles 1–4 (410 bp), 2–2 (240 bp), 3–5 (500 bp), 4–3 (335 bp) and 5–6 (595 bp). Samples showing one DNA fragment or one allele of 410 bp were considered as homozygote wild type and those with one DNA fragment at 240, 335, 500 or 595 pb were homozygote mutants. Samples presenting two DNA fragments with 410 bp and another one were considered as heterozygote (Table 2).

### Amplification of variable number of tandem repeats of *HP* gene

Genotyping the *HP* polymorphism was performed using two approaches: a PCR approach described by Koch *et al*. [38] and PCR-RFLP using two restriction enzymes to confirm results obtained by PCR [38].

The direct PCR approach consists of two separate PCR reactions with specific DNA fragment characterizing each genotype. Primers A/B (GAGGGGAGCTTGCCTTTCCATTG and GAGATTTTTGAGCCCTGGCTGGT) amplified DNA fragments of 1,757 bp and 3,481 bp for homozygote wild type (genotype Hp1/1) and homozygote mutant (genotype Hp2/2) respectively. Samples showing two DNA fragments at 1,757 bp and 3,481 bp were considered as heterozygote with genotype Hp1/2. Since the 3,481 bp fragment might not amplify due to lower efficiency of PCR for large fragments or sheared genomic DNA, the results were subsequently validated by a second amplification with primers C (CCTGCCTCGTATTAACTGCACCAT) and D (CCTGCCTCGTATTAACTGCACCAT), which amplify a specific DNA fragment of 349 bp for the Hp2 allele [38].

For each of these pairs of primers, the PCR reactions were carried out in a final volume of 25 μl containing 5–10 ng of DNA, 2.5 mM MgCl_2_, 0.2 mM of each dNTP, 20 pmol of each primer and 0.5 units of Taq polymerase (Qiagen). The amplification program was 95°C for 5 min followed by 35 cycles of 95°C for 60 s, 69°C for 90 s (primers A/B) or 60 s (primers C/D) and 72°C for 2 min. A final extension was done at 72° C for 5 min. The amplified products were separated by electrophoresis on a 2% agarose gel at 100 volts for 1 h30 min.

To confirm results (alleles of Hp1 and Hp2) obtained by PCR, the DNA fragments of 1757 bp and 3481 bp of primers A/B were digested with *Mls*I, while the fragment of 349 bp of primers C/D was digested with *Dra*I. Briefly, 10 μl of amplified DNA fragments of each of the primers set was digested with *Mls*I or *Dra*I as recommended by the supplier (Thermo Fisher). The digestion was done overnight at 37°C in the buffer 3.1 provided by the manufacturer. The digested products were separated by electrophoresis on a 2% agarose gel at 100 volts for 2 h 30 min.

### Polymorphism in *HLA-G* genes through the analysis of 14bp Indel

The polymorphism at 3’UTR (rs371194629) of HLA-G was evaluated by PCR as described by Castelli *et al*. [41]. PCR reactions were performed in a final volume of 25 μl containing 0.2 mM of each dNTP, 1.5 mM MgCl_2_, 20 pmol of each primer (HLA-G8F: TGTGAAACAGCTGCCCTGTGT and HLA-G8R: GTCTTCCATTTATTTTGTCTCT), 0.5 unit of *Taq* polymerase (Qiagen) and 5–10 ng of genomic DNA. The amplification program was 95°C for 5 min followed by 35 cycles. Each of these cycles was made up of 95°C for 45 s, 56°C for 45 s and 72°C for 1 min. A final extension was performed at 72° C for 5 min.

The amplified products were resolved by electrophoresis on 4% agarose gel for 4 hours at 100 volts. After this resolution, homozygote mutant and homozygote wild type genotypes were identified through DNA fragments of 345 bp and 359 bp for deletion (Del) and insertion (Ins) alleles respectively. For heterozygote genotype, two DNA fragments of 345 bp and 359 bp were expected.

### Statistical analysis

For this study we assumed an additive genetic model where two risk alleles of a SNP (homozygous) have twice the effect of one risk allele (heterozygous) [42]. Power calculation was undertaken using the PGA modeller package in MATLAB software [42]. For this package, the power was calculated by considering an odd ratio (OR) or relative risk (RR) >2 for loci with disease allele frequencies of 0.052–0.500 with 7 loci genotyped. Other factors taken into consideration include the disease prevalence estimated at <0.01 [43], the standard linkage parameter (r^2^) for Linkage disequilibrium (LD) of 0.7 [42], a type 1 error of 5% risk and sampling size. This later was estimated as described by Kasiulevicius et al. [44] using the independent case-control sampling size formula [44]. For this estimation, we assumed an expected exposure proportions in control of 0.20, a disease prevalence of < 0.01 [43] and a case-control ratio of 1:3. With the independent case-control sampling size formula, the sampling size to detect a real odds ratio or case exposure rate with power and two-sided type I error of 5% risk was 480 including 120 HAT cases and 360 controls.

Due to the heterogeneity of the study population formed by 19 ethno-linguistic subgroups (S1 Table) and its effect on the Hardy-Weinberg equilibrium and the risk that associations results might be bias by the stratified population rather than infections due to *T*. *b*. *gambiense*, the data were firstly stratified and analysed by three major ethno-linguistic groups (Bantu, Semi-Bantu and Baka). A second analysis was performed when 10 ethno-linguistic subgroups were pooled into two groups on the basis of similarities in language spoken and their geographical proximity [45, 46]. These two groups include the Beti-Fang (Bulu, Eton, Fan, Iyassa, Kwasse, Maabi, Mvae and Ngoumba ethno-linguistic subgroups) and Wovea (Douala and Bassa ethno-linguistic subgroups) (S2 Table). To confirm results generated on the population that was stratified into major ethno-linguistic groups and two pooled ethno-linguistic subgroups, 11 ethno-linguistic subgroups derived from this stratified population including the Bamilike, Bassa, Douala, Eton, Fan, Iyassa, Kwasse, Maabi, Mvae, Mundani and Baka (S2 Table) were further separately analysed with the fisher exact test at midpoint.

Hardy-Weinberg analysis was run not only on the entire population, but also on each individual ethno-linguistic group or subgroup in order to observe the effect the population heterogeneity on HWE, the power of our study and association results. Ethno-linguistic groups or subgroups with HWE p-value deviation and less than 10 individuals or no informative data at a locus were removed for subsequent analyses.

The Cochran-Mantel-Haenszel (CMH) test implemented in PLINKv1.9 package [47] was performed with the allelic frequencies because this test can only be done with binary vars. Used as an extension of the chi-square test, the CMH test enabled to estimate the odds ratio and 95% confidence interval across the stratified populations represented here by ethno-linguistic subgroups. Using these later as covariates, it enabled to test for associations between alleles and the probability to be infected by *T*. *b*. *gambiense* and develop HAT within each ethno-linguistic subgroup. However, the CMH2 test, also implemented in PLINKv1.9 package, was used to determine if there were significant differences in the allele frequencies between different ethno-linguistic groups or subgroups. Data were visualised with R/Rstudio version 3.3.2 (2016-10-31). Results of multiple tests were adjusted by the Bonferroni correction which assumes that each of the statistical tests is independent. The significance of genotype and allele frequency differences between cases and controls within each ethno-linguistic group or subgroup were obtained and confirmed with the Fisher exact test for 2x2 contingency table.

A meta-analysis was performed on samples from ethno-linguistic subgroups that were in HWE and that showed significant association with the Fisher exact test. This was done not only on each major ethno-linguistic group, but also on the Beti-Fang and Wovea ethno-linguistic groups and the 10 ethno-linguistics subgroups mentioned above.

## Results

### Study designed

For this study, a total of 323 individuals including 73 (22.60%) HAT cases and 250 (77.40%) controls were analysed. The 323 individuals belonged to 19 different ethno-linguistic subgroups (S1 Table). Of these 323 individuals, 211 (65.33%) were Bantu, 84 (26.01%) semi-Bantu, 21 (6.50) Baka and 7 (2.17%) Sudano-Sao (S1 Table). The mean age (range) was 45.75±5 (14–91) for HAT cases and 37.58±5 (9–88) for controls. No significant difference (t = -0.206, *P* = 0.837) was observed between the age of controls and HAT cases. The overall sex ratio (male/female) was 1.006 with 49.85% (161/323) of female and 50.15% (162/323) of male.

For this study, we genotyped one polymorphism in each of the seven genes (*IL1A*, *IL1RN*, *IL4RN*, *IL6*, *HP*, *HPR*, and *HLA-G*) in 73 cases and 250 controls collected from 19 ethno-linguistic subgroups from three Cameroonian HAT foci.

With LD r^2^ of 0.7, a disease prevalence of <0.01, the disease allele frequencies of 0.052–0.500 for 7 loci genotyped, and a sampling size of 323 individuals including 73 HAT cases and 250 controls, the power of this study was estimated at 82%.

### Genes and loci selected and genotyped

Seven loci containing 3 SNPs, 3 VNTRs and one indel were tested from 7 candidate genes. The polymorphism at each of these loci was investigated on 323 samples containing 73 HAT cases and 250 controls from three HAT foci of southern Cameroon. From 323 samples that were analyses at different loci, more than 94% were successfully genotyped at each of the 7 loci. At all loci except *HLA-G*, the reference allele was at higher frequency than the alternate allele. The genotypes 1A1A (allele 1–4: 410 bp), 1A4A (allele 4–3: 335 bp) and 1A3A (allele 3–5: 500 bp) were identified for *IL1RN* gene while the genotypes 2A2A (allele 2–2: 240 bp) and 5A5A (allele 5–6: 595 bp) or their heterozygote genotypes combinations were absent in our studied population.

For the 318 samples that were successfully genotyped at the SNPs rs1800794 of *IL1A*, 97 (30.5%), 179 (56.3%) and 42 (13.2%) were respectively heterozygote, homozygote wild-type and homozygote mutant. At SNP rs1554606 of *IL6*, 98.8% (319/323) of samples were successfully genotyped: 41.4% (132/319) were heterozygote while 49.2% (157/319) and 9.4% (30/319) were homozygote wild-type and mutant respectively. At SNP rs1697370 of *HPR*, 99.4% (321/323) of samples were genotyped: 33.3% (107/321) were heterozygote whereas 57.0% (183/321) and 9.7% (31/321) were homozygote wild-type and mutant respectively.

Regarding the VNTR, 322 (99.7%: 322 /323) samples were successfully genotyped for the genes HP and *IL4RN*. For *HP*, 180 (55.9%: 180/322) samples were heterozygote while 82 (25.5%: 82/322) and 60 (18.6%: 60/322) were homozygote wild-type and mutant respectively. For *IL4RN* at locus rs79071878, 176 (54.7%: 176/322), 77 (23.9%: 77/322) and 69 (21.4%: 69/322) were heterozygote, homozygote wild-type and homozygote mutant respectively. At locus rs2234663 of *IL1RN*, 99.1% (320/323) of samples were genotyped: 89.1% (285/320) were homozygote wild-type and the remaining was heterozygote.

For the indel HLA-G at locus rs371194629, 99.1% (320/323) of samples were genotyped and 55.9% (179/323), 18.1% (58/320) and 25.9% (83/320) were heterozygote, homozygote wild-type and homozygote mutant respectively.

### Association study performed on the whole population

Allele and genotype frequencies of all cases were compared with those of all controls at all loci using chi-squared tests. No significant difference was observed for the 14 bp indel located at rs371194629 of *HLAG*, the SNPs rs1554606 and rs1697370 of *IL6* and *HPR* respectively and the VNTRs of *IL4RN* and HP (Table 3). However, a significant increased risk to be infected by *T*. *b*. *gambiense* and develop HAT was observed with the TT genotype in *IL1A* gene with an OR of 2.938 (CI_95_ [1.56–3.89]) and a *P value* of 0.0010. In addition, the genotype 1A/3A located at locus rs2234663 of *IL1RN* VNTR with an OR of 2.71 (CI_95_ [0.97–7.58]) and a *P value* of 0.0012 was also associated with an increased risk of getting *T*. *b*. *gambiense* infections and develop HAT. However, the frequencies of this genotype were low in both cases (8.3%) and controls (3.6%) (Table 3) and this observation should be considered provisional until replicated in larger studies because only 7 cases and 9 controls were enrolled in the analyses. The observed differences in the allelic frequencies distribution (S3 Table) and their corresponding *p values* for the 7 loci within *IL1A*, *IL6*, *HP*, *HPR*, *IL1RN*, *IL4RN* and *HLA-G* were deduced from genotypes data contained in Table 3. Although *IL1A* seems to be associated with an increased risk of getting *T*. *b*. *gambiense* infections and develop HAT, the allele frequencies were not in Hardy-Weinberg equilibrium (HWE) (0.007). However, when the population was stratified into ethno-linguistic subgroups or major ethno-linguistic groups, the allele frequencies were in HWE for most loci genotyped as shown in S4 Table. These results indicate that the heterogeneous nature of the studied population, formed by several ethno-linguistic subgroups, has an impact on the HWE. Due to these variations and the deviation of HWE in the entire population, additional analyses were performed with the Cochran-Mantel-Haentszel test (CMH) that takes into account the population stratification. For these analyses, the population was stratified on the basis of ethno-linguistic groups and subgroups.

**Table 3:** Genotype frequency distribution for gene polymorphisms within the global Cameroon population.

Loci	Genotypes	Case (%)	Control (%)	P-value	OR (95%CI)	BONF	X^2^	HWE
**INDEL**	***HLAG*: rs371194629 (Case = 72, Control = 248)**							
	Ins/Ins	11 (15.28)	47 (18.88)	-	-	-	-	0.0749
	Del/Ins	41 (56.94)	138 (55.42)	0.360	1.27 (0.31–3.98)	1	0.837	
	Del/Del	20 (27.78)	63 (25.30)	0.758	1.36 (0.39–4.98)	1	0.554	
**VNTR**	***IL4RN*: rs79071878 (Case = 73, Control = 249)**							
	R1/R1	12(16.44)	65 (26.12)	-	-	-	-	0.3739
	R1/R2	44 (60.27)	132 (53.01)	0.661	1.81 (0.83–4.05)	1	0.193	
	R2/R2	17 (23.29)	52 (20.88)	0.234	1.77 (0.70–4.72)	1	2.903	
	***HP* (Case = 72, Control = 250)**							
	Hp1/1	14(19.45)	68 (27.20)	-	-	-	-	0.0965
	Hp1/2	42(58.33)	138 (55.20)	0.377	1.48 (0.59–2.45)	1	0.779	
	Hp2/2	16(22.22)	44 (17.60)	0.357	1.77 (0.88–4.70)	1	2.059	
	***IL1RN*: rs2234663 (Case = 72, Control = 248)**							
	1A/1A	60(84.72)	209(84.27)	**-**	-	-	-	
	1A/4A	5(6.95)	30(12.10)	0.217	0.58 (0.21–1.56)	1	1.521	1
	**1A/3A**	**7(8.33)**	**9(3.63)**	**0.0012**	**2.71 (0.97–4.08)**	**0.0084**	**21.727**	**1**
**SNP**	***IL1A*: rs1800794 (Case = 72, Control = 246)**							
	CC	31 (43.06)	148 (60.16)	-	-	-	-	0.007
	TC	25 (34.72)	72 (29.27)	0.390	1.66 (1.15–4.92)	1	1.455	
	**TT**	**16 (22.22)**	**26 (10.57)**	**0.0010**	**2.94(1.56–3.89)**	**0.0072**	**9.166**	
	***IL6*: rs1554606 (Case = 73, Control = 248)**							
	TT	37 (50.68)	120 (48.39)	-	-	-	-	0.6489
	TG	26 (35.62)	108 (43.55)	0.734	0.66 (0.59–1.40)	1	0.115	
	GG	10 (13.70)	20 (8.06)	0.152	1.21 (079–3.37)	1	3.766	
	***HPR*: rs1697370 (Case = 73, Control = 248)**							
	TT	41 (56.16)	142 (57.26)	-	-	-	-	0.1322
	TC	22 (30.14)	85 (34.27)	0.684	0.94 (0.63–2.04)	1	0.166	
	CC	10 (13.70)	21 (8.47)	0.387	1.17 (0.85–3.53)	1	1.899	

(%): genotype frequencies; P-value: Nominal P unadjusted asymptotic probability value; OR: odds ratio; X^2^: Chi-square probability value; CI: Confidence Interval at 95%; N: sample size (cases/controls); SNP: Single Nucleotide Polymorphism; VNTR: Variable Number Tandem Repeats; INDEL: Insertion and Deletion; *HP*:Haptoglobin; *HPR*: Haptoglobin related protein; *IL*: Interleukins; *HLA*: Human Leukocyte Antigen

### Association study considering the stratified populations

After stratification of our study population into three major ethno-linguistic groups (Bantu, Semi-Bantu and Baka), the observed allelic frequencies were all in Hardy–Weinberg equilibrium within each ethno-linguistic group; suggesting random genetic exchange within each of the major ethno-linguistic groups. Data of S4 Table shows detailed results of HWE values when the population was structured into ethno-linguistic groups and in pooled ethno-linguistic subgroups.

The Cochran-Mantel-Haentszel test (CMH) was used to test the associations between the allele frequencies and the probability to be infected by *T*. *b*. *gambiense* and develop HAT. This test estimates an odds ratio and 95% confidence interval across the population using ethno-linguistic subgroups as covariant. Data of CMH test reported in Table 4 considered only 305 individuals (69 HAT cases and 236 controls) of three major ethno-linguistic groups. The null hypothesis of the Cochran-Mantel-Haenszel (CMH) test is that allele frequencies are the same in cases and controls and do not differ between populations. With the CMH test, the minor allele T of rs1800794 in *IL1A* which is located in the promoter region was significantly associated (unadjusted P = 0.0012, X^2^ = 30.01, adjusted P = 0.009) with an increased risk to be infected by *T*. *b*. *gambiense* and develop this infection (Table 4). Its OR of 2.066 (CI_95_ [1.33–3.20]) and P value of 0.009 indicate higher frequencies in cases compared to controls.

**Table 4:** Cochran-Mantel-Haenszel (CMH) association analysis results within the Cameroonian population.

Loci	Gene	rsid	Alleles	MAF	P-value	BONF	CHISQ	^a^P-value	^a^BONF	^a^CHISQ	OR (95%CI)
**SNP**	***IL1A***	rs1800794	*T **C	0.285	0.0012	0.009	10.45	0.368	1	30.01	2.066(1.33–3.20)
	***IL6***	rs1554606	*G **T	0.290	0.6117	1	0.258	0.654	1	17.95	0.892(0.57–1.39)
	***HPR***	rs1697370	*C **T	0.262	0.1498	1	2.074	0.016	0.115	37.08	1.395(0.88–2.20
**VNTR**	***HP***	-	*Hp2 **Hp1	0.483	0.0968	0.677	2.758	0.803	1	15.38	1.411(0.94–2.12)
	***IL4RN***	rs79071878	*2R **1R	0.487	0.2673	1	1.231	0.770	1	15.99	1.26(0.84–1.89)
	***IL1RN***	rs2234663	*3A *4A **1A	0.051 0.0487	0.0859 0.4976	1 1	2.95 0.46	0.693 0.260	1 1	7.3416 24.72	2.651(0.86–8.15) 0.658(0.20–2.14)
**INDEL**	***HLA-G***	rs371194629	*Del **Ins	0.466	0.9599	1	0.003	0.361	1	22.69	1.011(0.67–1.52)

P-value: Nominal CMH P unadjusted asymptotic probability value

*: minor allele

**: major allele: CHISQ: Chi-square probability value; OR: odds ratio; BONF: Bonferroni adjusted asymptotic p value, MAF Minor allele frequency

^a^: Cochran-Mantel-Haenszel for homogeneity of association across clusters (using the cmh2 test in plink), rsid: reference SNP identification code: SNP: Single Nucleotide Polymorphism; VNTR: Variable Number Tandem Repeats; INDEL: Insertion and Deletion.

The null hypothesis of the CMH2 test is that allele frequencies are the same in each population. The CMH2 test indicated that there was no significant difference in allele frequencies between populations (P = 0.368). The Bantu major ethno-linguistic subgroups were pooled (Beti-Fang: Bulu, Eton, Fan, Iyassa, Kwasse, Maabi, Mvae, and Ngoumba; and Wovea: Bassa and Douala ethno-linguistic subgroups) into two groups (S4 Table) in order to trace and spot which of these subgroups was at the centre of this effect. With CMH test, the minor allele T of rs1800794 in *IL1A* remains significantly (unadjusted P = 0.0005, X^2^ = 11.99, adjusted P = 0.004) associated with an increased risk of getting *T*. *b*. *gambiense* and develop this infection in the Bantu major ethno-linguistic group. Its OR of 2.32 (CI_95_ [1.44–3.37]) and a P value of 0.0005 (S2 Table) indicates higher frequencies of the allele T in cases compared to controls. After pooling some ethno-linguistic subgroups (S2 Table), the minor allele T of rs1800794 in *IL1A* with an OR of 2.40 (CI_95_ [1.41–4.10]) and an adjusted P value of 0.009 remains significantly associated with an increased risk of getting *T*. *b*. *gambiense* infections and develop HAT within the Beti-Fang ethno-linguistic groups.

The HP2 minor allele of *HP* seems to be also significantly (unadjusted P = 0.0015, X^2^ = 5.90, adjusted P = 0.011; OR: 3.68 (CI_95_ [1.23–8.33])) associated with an increasing risk of getting *T*. *b*. *gambiense* infections and develop HAT within the Bassa and Douala ethno-linguistic subgroups (S2 Table). For the other genes, no significant difference was observed in the association studies as reported on whole population (Table 4).

To confirm results obtained on the stratified populations and see the impact of heterogeneous population or different ethno-linguistic groups and subgroups on the association between gene polymorphism and the risk to be infected by *T*. *b*. *gambiense* and develop HAT, meta analyses were performed on the basis of the three major ethno-linguistic groups and 11 ethno-linguistic subgroups.

### Meta analyses on the basis of ethno-linguistic groups

Of the 323 individuals belonging to the 19 different ethno-linguistic subgroups used in this study (S1 Table), 75 of them were excluded due to small population sample size (i.e less than 10 individuals), small HWE *P values* (S5 Table) and or loci that were not informative (low genotypes and allelic frequencies). For subsequent analyses, 249 individuals belonging to the three major ethno-linguistic groups and 11 ethno-linguistic subgroups (Bamilike, Bassa, Douala, Eton, Fan, Iyassa, Kwasse, Maabi, Mvae, Mundani and Baka) were considered for association analysis (S5 Table). The observed allelic frequencies were all in Hardy–Weinberg equilibrium within the ethno-linguistic subgroups; suggesting random genetic exchange within these ethno-linguistic groups and subgroups.

Results of meta-analysis confirmed the significant (P = 0.0017, OR = 2.305) association previously reported for SNP rs1800794 of *IL1A* (Table 5). Its OR of 2.305 (CI_95_ [1.29–3.25]) and a P value of 0.0017 confirms the higher frequencies of allele T in cases compared to controls. The absence of significant association at different loci of other genes was also confirmed by the meta-analysis. Results generated by meta analyses on the 11 ethno-linguistic subgroups were consistent with those of the CMH test on the populations that were stratified into three major ethno-linguistic groups and pooled ethno-linguistic subgroups.

**Table 5:** Meta-analysis of the Fisher exact test results within the 11 ethno-linguistic groups clusters.

CHR	GENE	BP (GRch37)	rsid	Alleles	N	P	P(R)	OR	Q	I
**2**	*IL1A*	113543273	rs1800794	*T **C	8	0.0017	0.0017	2.305	0.9176	0.00
**5**	*IL4RN*	132680584	rs79071878	*2R **1R	9	0.1299	0.1299	0.6923	0.9781	0.00
**6**	*HLA-G*	29830805	rs371194629	*Del **Ins	9	0.7525	0.7525	1.0793	0.8060	0.00
**7**	*IL6*	154426970	rs1554606	*G **T	8	0.9748	0.9361	1.0082	0.2183	26.37
**16**	*HPR*	35339932	rs1697370	*C **T	8	0.6692	0.6692	0.8867	0.8810	0.00
**16**	*HP*	-	-	*Hp2 **HP1	10	0.09482	0.09482	0.6690	0.6859	0.00

SNP: single nucleotide polymorphism, BP (GRch37): base-pair location (Reference human genome build version); rsid: reference SNP identification code

*: minor allele

**: major allele; P: fixed-effects meta-analysis asymptotic probability value; P(R): random-effects meta-analysis asymptotic probability value; OR: fixed-effects odds ratio estimate; Q: probability value for Cochrane’s Q statistics; I: I^2 heterogeneity index (0–100); CHR: Chromosome; N: number of valid studies for this.

## Discussion

In this study, 7 polymorphisms within 7 genes were investigated for their association with *T*. *b*. *gambiense* infection in southern Cameroon. The most important observation is that the minor allele (T) of *IL1A* genes could influence the infections due to *T*. *b*. *gambiense* in HAT foci of southern Cameroon. Indeed, when the 19 ethno-linguistic subgroups were grouped together and considered as one population, *IL1A* seems to be associated with an increased risk of getting *T*. *b*. *gambiense* infections and develop HAT, but the allele frequencies were not in HWE (0.007). This deviation of HWE could be due to the heterogeneity of our studied population formed by 19 ethno-linguistic subgroups with some genetic differences. This heterogeneity led to a deviation of HWE resulting probably from the Wahlund effect [48] that is caused by the variance in allele frequency among subpopulations [48–50]. Indeed, in rural areas were HAT is often found, the populations are grouped according to their ethno-linguistic subgroups with very few probabilities of marriage between people from different sub-groups. This social behaviour may lead to Wahlund effect that has an impact on HWE due to the lack of genetic exchange between populations of different ethno-linguistic subgroups. In consequence, an increase of inbreeding rate, a strong genetic drift and a decrease of the genetic diversity could be observed within and between these populations [51–53]. The small sample size of some ethno-linguistic subgroups could also increase the inbreeding effect on the high variance of allele frequencies (S4 Table). The heterogeneous structure of our population may impale a strong genetic drift that changes the gene ratio of population in a random manner. Moreover, it has been reported that the genotyping error impaled by the methods used could increase the heterozygote frequency and the observation of some mutant alleles [54–57]. These hypotheses are strengthened by the differences observed for the values of HWE within and between different ethno-linguistic subgroups (S4 Table). All these factors may induce biases in the association studies and consequently, a reduction of the power of this study.

Following stratified (Table 4) and meta analyses (Table 5), the minor allele T of rs1800794 SNP of *IL1A* gene was significantly associated with an increased risk of getting *T*. *b*. *gambiense* infections and develop HAT in major ethno-linguistic groups and subgroups of southern Cameroon. Our observations of *IL1A* were not consistent with results for the same SNP in a family based linkage study in DRC [6] which showed no significant differences in transmission rates of the C and T alleles to affected children (p = 0.56). The discrepancy between the two sets of results could be due to the study design, the small sample size (73 cases belonging to several ethno-linguistic subgroups) and the genetic differences between the DRC and Cameroon populations. Our study was a classical case-control study involving controls and HAT patients from different families while in DRC, a family base study was performed with HAT cases and controls from the same family [6], Indeed, *IL1* participates in macrophage activation during early *T*. *b*. *gambiense* infection in mice [15] and plays a key role in the recruitment of leukocytes into the CNS through the blood-brain barrier during CNS infection [16, 17]. Due to the fact that HAT cases and controls were matched according to sex, village and their activities, they are likely subjected to equal levels of tsetse bites. Assuming their equal exposition to tsetse bites, we can speculate that the *IL1a* variant might render some individuals refractory to *T*. *b*. *gambiense* infections. This hypothesis may increase the likelihood that some infected HAT cases might self-cure as reported in West Africa [5]. It may also increase the likelihood that some seropositive individuals could be parasitologically negative. Such individuals could be carriers of low *T*. *b*. *gambiense* load that are below the detection threshold of parasitological techniques commonly used during medical surveys. Although well confirmed and phenotyped seropositive individuals were not included in this study, it is important to mention that an association has been reported between gene polymorphism and the progression of sleeping sickness from latent infection to active disease [13].

The 1A/3A genotype (P = 0.0012 and OR of 2.71) of *IL1RN* rs2234663 VNTR was associated with an increasing risk of getting *T*. *b*. *gambiense* infections and develop HAT. Although the allelic frequency in cases and controls is low, the results obtained indicate an increasing risk to be infected and develop *T*. *b*. *gambiense* infection in HAT foci of southern Cameroon. *IL1RN* is located in the chromosome 2q14 [58, 59] with an 86 bp VNTR polymorphism in its second intron [60, 61]. *IL1RN* polymorphisms are also closely associated with the regulation of IL1B activity that enhance and stimulate parasite interaction and neutralisation via the complement pathways [62, 63]. It has been proposed that individuals with the *IL1RN* 2A/2A genotype have elevated levels of circulating *IL1B*. The increased *IL1B* levels result in a prolonged inflammatory response and increase the risk of pyloric gastric disease [36]. This elevated level of *IL1B* also enhances immune-modulating and stimulating effects of the IL1 family gene on some immune response components [64]. *IL1* family genes have been shown to enhance immune-modulating and stimulating effects on the TLF components and pro/inflammatory immune response activities during HAT infection [29, 30]. Although this is the first observation of an association between *IL1RN* and *T*. *b*. *gambiense* infections, associations have been already reported between *IL1RN* variants and other diseases such as keratoconus in a Korean population [65], *H*. *pylori* gastric [36] and periodontitis disease [64] in the Brazilian population, and hepatitis and primary biliary cirrhosis in the Chinese population [66].

Our findings suggest an association between *IL1* family genes (*IL1RN* and *IL1A*) and the risk of getting *T*. *b*. *gambiense* infections and develop HAT in southern Cameroon. Although the mechanism leading to this infection is not well understood, the hypothesis is that the blood-brain barrier permeability is modified due to the presence, in blood and/or in CNS, of inflammatory mediators such as *IL1*, *IL6* and *TNFA* [67]. It has been shown *in vitro* that *T*. *b*. *gambiense* induces the synthesis of inflammatory and pro-inflammatory cytokines like *IL6* and *IL1* from the bone marrow endothelial cells [68]. The involvement of these cytokines, whose level increases in the cerebrospinal fluid (CSF) during *T*. *b*. *gambiense* infection and decreases after treatment, has been confirmed [69]. *IL1A* and *IL1RN* are important immunologic regulators that compete with other *IL1* family members for the *IL1* receptor and act as negative regulators with anti-inflammatory effects [70, 71] and also in differential modulation of *IL1* activity [72]. These variants are in linkage disequilibrium with other unidentified and identified variants in the *IL1* gene family and it remains to be determined which are the functional polymorphism(s).

The minor allele HP2 with a P value of 0.0015 and an OR of 3.68 is associated with an increased risk of getting *T*. *b*. *gambiense* infections and develop HAT in Bassa and Douala ethno-linguistic subgroups (S2 Table). These results are in line with those reported in South American where an increasing risk effect of Hp2/2 genotype and HP2 allele was observed for *T*. *cruzi* infections [73, 74]. In gastric cancer, similar results were observed for Hp2/2 genotype [75]. However, Hp2/2 genotype and Hp2 allele were suggested to be protective against infections due to *Plasmodium falciparum* during severe malaria [34]. These results indicate the involvement of Hp2/2 and HP2 in many infectious diseases. The geographical differences in the allele frequency of Hp2 could explain its association and involvement with different susceptibility to infectious diseases [76]. Indeed, *HP* is involved in scavenging haem from lysed red blood cells and trypanosomes cause extensive lysis of red cells, leading to a decline in circulating haem. A decline in the expression of the haptoglobin receptor on macrophages indicates earliest detectable sign of infection of mice with *T*. *congolense*. This expression of haptoglobin receptor (*Cd163*) declined tenfold by day 3 post infection before there was a detectable parasitaemia [77].

Our results showing no significant association for SNPs rs1554606 of *IL6* and 14 bp indel at rs371194629 of *HLA-G* do not corroborate results reported in DRC and Guinea [6, 7, 13]. For the same genes, but at different loci, Courtin *et al*. [7] showed that the T allele at rs2069849 of *IL6* seems to significantly (Bonferroni corrected P = 0.04) decrease the risk of getting *T*. *b*. *gambiense* infections in the DRC while Kabore et al. [13] reported an association between the allele *A* at rs1818879 locus of *IL6* with the low risk of HAT progression in Guinea. The discrepancies between these results could be due to insufficient linkage between our SNPs and those (rs2069849 and rs1818879) genotyped by other authors, the genetic differences between the DRC, Guinea and the Cameroon populations, the genotyping method and the sample size. The study designs also differed because we used a case control approach while Courtin *et al*. [6] used a family-based design and our small sample size has impact on the power of this study.

The results discussed above for *IL6*, *IL4* and *HLA-G* should be considered with caution because of the heterogeneity of the studied population that induced some smaller sample sizes and limited the power of this study. Despite the efforts undertaken through several large-scale field surveys conducted in three HAT foci, we were only able to collect a relatively small number of 73 HAT cases. As already indicated in our previous publication [11], our power calculations indicated that effects of the sizes could be detected with our relatively small number of samples. However, larger cohorts of well phenotyped cases and controls may be required to confirm our observations. Although the present data is only suggestive of an association, the finding of suggestive associations in multiple populations may increase the probability that these are genuine associations with sleeping sickness.

### Conclusion

This study revealed that one SNP (rs1800794) of *IL1A* and one VNTR (rs2234663) of *IL1RN* were associated with an increased risk to be infected by *T*. *b*. *gambiense* and develop HAT in inhabitants of sleeping sickness foci of southern Cameroon. The minor allele (T) of SNP rs1800794 of *IL1A* gene and the genotype 1A3A of *IL1RN* rs2234663 VNTR seem to increase the risk of getting *T*. *b*. *gambiense* infections and develop HAT in southern Cameroon. Results of this study show that the association between host genetic determinants or gene polymorphisms and the risk to be infected by *T*. *b*. *gambiense* and develop HAT may vary with the heterogeneity of the studied populations.

## Supporting information

S1 Table Ethno-linguistic composition of HAT cases and controls of the studied population. (DOCX)

S2 Table The observed allelic frequencies differences distribution within Cameroon population. (DOCX)

S3 Table Cochran-Mantel-Haenszel (CMH) association analysis results within the major ethnic groups and pooled ethno-linguistic subgroups within the population. (DOCX)

S4 Table Variations of HWE values according to loci and ethno-linguistic groups and subgroups. (DOCX)

S5 Table Fisher analysis results within three major ethnic groups and 10 ethno-linguistic subgroups. (DOCX)
