# Supplementary material for: Association between IL1 gene polymorphism and human African trypanosomiasis in populations of sleeping sickness foci of southern Cameroon
Source: PLoS Negl Trop Dis. 2019 Mar 25;13(3):e0007283. doi: 10.1371/journal.pntd.0007283 (PMC6448947; doi:10.1371/journal.pntd.0007283)
Supplement: S1 Table — (DOCX) [file pntd.0007283.s001.docx]

**S1Table:** Ethno-linguistic composition of HAT cases and controls of the studied population

| Major ethnic groups | Ethno-linguistic subgroups | Cases | Control | Total (cases/controls) |
| --- | --- | --- | --- | --- |
| Bantu | Ngoumba | 14 | 32 | 211 (61/150) |
|  | Mvae | 06 | 09 |  |
|  | Fan | 01 | 12 |  |
|  | Maabi | 04 | 09 |  |
|  | Iyassa | 08 | 23 |  |
|  | Kwasse | 06 | 08 |  |
|  | Bassa | 12 | 28 |  |
|  | Eton | 09 | 13 |  |
|  | Douala | 01 | 10 |  |
|  | Bulu | 00 | 06 |  |
| Semi-Bantu | Bamileke | 04 | 12 | 85 (07/78) |
|  | Banyangi | 00 | 12 |  |
|  | Wimbum | 00 | 02 |  |
|  | Tika | 00 | 01 |  |
|  | Mundani | 03 | 50 |  |
|  | Modelle | 00 | 01 |  |
| Sudano-sao | Foulani | 00 | 01 | 06 (00/06) |
|  | Moudan | 00 | 05 |  |
| Baka | Pigmies | 05 | 16 | 21 (05/16) |
| Total | | 73 | 250 | 323 (73/250) |
