# Supplementary material for: Association between IL1 gene polymorphism and human African trypanosomiasis in populations of sleeping sickness foci of southern Cameroon
Source: PLoS Negl Trop Dis. 2019 Mar 25;13(3):e0007283. doi: 10.1371/journal.pntd.0007283 (PMC6448947; doi:10.1371/journal.pntd.0007283)
Supplement: S2 Table — (DOCX) [file pntd.0007283.s002.docx]

**S2 Table:** Cochran-Mantel-Haenszel (CMH) association analysis results within the major ethnic groups and pooled ethno-linguistic subgroups within the population

| Eth.GP | Loci | Gene | rsid | Alleles | MAF | P-value | BONF | CHISQ | OR (95%CI) |
| --- | --- | --- | --- | --- | --- | --- | --- | --- | --- |
| Bantu | SNP | ***IL1A*** | rs1800794 | *T  **C | 0.278 | **0.0005** | **0.0037** | **11.99** | **2.32 (1.44-3.74)** |
|  |  | ***IL6*** | rs1554606 | *G  **T | 0.317 | 0.8128 | 1 | 0.056 | 0.94 (0.60-1.50) |
|  |  | ***HPR*** | rs1697370 | *C  **T | 0.229 | 0.3014 | 1 | 1.056 | 1.30 (0.79-2.14) |
|  | VNTR | ***HP*** | - | *Hp2  **Hp1 | 0.461 | 0.2088 | 1 | 1.58 | 1.32 (0.86-2.04) |
|  |  | ***IL4RN*** | rs79071878 | *2R  **1R | 0.493 | 0.3291 | 1 | 0.953 | 0.93 (0.60-1.45) |
|  |  | ***IL1RN*** | rs2234663 | *3A  *4A  **1A | 0.034  0.029 | 0.3516  0.5835 | 1  1 | 0.8678  0.361 | 1.69 (0.56-5.06)  0.67 (0.0.17-2.67) |
|  | INDEL | ***HLA-G*** | rs371194629 | *Del  **Ins | 0.468 | 0.7635 | 1 | 0.091 | 1.25 (0.80-1.94) |
| Semi  Bantu  Semi  Bantu | SNP | ***IL1A*** | rs1800794 | *T  **C | 0.279 | 0.5545 | 1 | 0.349 | 1.46 (0.42-4.94) |
|  |  | ***IL6*** | rs1554606 | *G  **T | 0.266 | 0.4 | 1 | 0.711 | 0.51 (0.10-2.49) |
|  |  | ***HPR*** | rs1697370 | *C  **T | 0.329 | 0.2804 | 1 | 1.165 | 1.94 (0.58-6.43) |
|  | VNTR | ***HP*** | - | *Hp2  **Hp1 | 0.487 | 0.3903 | 1 | 0.738 | 1.69 (0.51-5.60) |
|  |  | ***IL4RN*** | rs79071878 | *2R  **1R | 0.468 | 0.2033 | 1 | 1.618 | 2.14 (0.66-6.93) |
|  |  | ***IL1RN*** | rs2234663 | *3A  *4A  **1A | NA  0.1139 | NA  0.7566 | NA  1 | NA  0.096 | NA  0.69 (0.07-6.59) |
|  | INDEL | ***HLA-G*** | rs371194629 | *Del  **Ins | 0.475 | 0.4585 | 1 | 0.55 | 1.55 (0.49-4.97) |
| Baka | SNP | ***IL1A*** | rs1800794 | *T  **C | 0.368 | 0.0628 | 0.439 | 3.403 | 0.14 (0.02-1.32) |
|  |  | ***IL6*** | rs1554606 | *G  **T | 0.158 | 0.5163 | 1 | 0.421 | 0.51 (0.06-4.43) |
|  |  | ***HPR*** | rs1697370 | *C  **T | 0.342 | 0.981 | 1 | 0.001 | 1.02 (0.19-5.41) |
|  | VNTR | ***HP*** | - | *Hp2  **Hp1 | 0.474 | 0.7694 | 1 | 0.086 | 1.25 (0.29-5.47) |
|  |  | ***IL4RN*** | rs79071878 | *2R  **1R | 0.447 | 0.6755 | 1 | 0.175 | 0.71 (0.16-3.24) |
|  |  | ***IL1RN*** | rs2234663 | *3A  *4A  **1A | NA  0.026 | NA  0.2207 | NA  1 | NA  1.5 | NA  NA |
|  | INDEL | ***HLA-G*** | rs371194629 | *Del  **Ins | 0.316 | 0.9197 | 1 | 0.01 | 0.92 (0.19-4.5) |
| Beti-Fang | SNP | ***IL1A*** | rs1800794 | *T  **C | 0.301 | **0.0012** | **0.009** | **10.44** | **2.40 (1.41-4.10)** |
|  |  | ***IL6*** | rs1554606 | *G  **T | 0.317 | 0.6846 | 1 | 0.165 | 1.11 (0.66-1.87) |
|  |  | ***HPR*** | rs1697370 | *C  **T | 0.247 | 0.3863 | 1 | 0.750 | 1.28 (0.73-2.25) |
|  | VNTR | ***HP*** | - | *Hp2  **Hp1 | 0.462 | 0.9068 | 0.677 | 0.013 | 1.03 (0.63-1.69) |
|  |  | ***IL4RN*** | rs79071878 | *2R  **1R | 0.468 | 0.6582 | 1 | 0.196 | 1.12 (0.68-1.84) |
|  |  | ***IL1RN*** | rs2234663 | *3A  *4A  **1A | 0.051  0.039 | 0.0859  0.5835 | 1  1 | 2.95  0.46 | 2.651(0.86-8.15)  0.67 (0.17-2.67) |
|  | INDEL | ***HLA-G*** | rs371194629 | *Del  **Ins | 0.452 | 0.9709 | 1 | 0.001 | 0.99 (0.60-1.64) |
| Wovea | SNP | ***IL1A*** | rs1800794 | *T  **C | 0.204 | 0.2032 | 1 | 1.619 | 2.01 (069-5.81) |
|  |  | ***IL6*** | rs1554606 | *G  **T | 0.316 | 0.2167 | 1 | 1.526 | 0.51 (0.18-1.48) |
|  |  | ***HPR*** | rs1697370 | *C  **T | 0.174 | 0.5617 | 1 | 0.337 | 1.37 (0.46-4.05) |
|  | VNTR | ***HP*** | - | *Hp2  **Hp1 | 0.459 | **0.0015** | **0.011** | **5.9** | **3.68 (1.23-8.23)** |
|  |  | ***IL4RN*** | rs79071878 | *2R  **1R | 0.429 | 0.2136 | 1 | 1.547 | 0.53 (0.20-1.40) |
|  |  | ***IL1RN*** | rs2234663 | *3A  *4A  **1A | 0.101  0 | 0.359  NA | 1  NA | 1.95  NA | NA  NA |
|  | INDEL | ***HLA-G*** | rs371194629 | *Del  **Ins | 0.48 | 0.5784 | 1 | 0.309 | 1.3 (0.52-3.25) |

P-value: Nominal CMH P unadjusted asymptotic probability value; *: minor allele; **: major allele: CHISQ: Chi-square probability value; OR: odds ratio; BONF: Bonferroni adjusted asymptotic p value, MAF Minor allele frequency; ^a^: Cochran-Mantel-Haenszel for homogeneity of association across clusters (using the cmh2 test in plink), SNP: Single Nucleotide Polymorphism; VNTR: Variable Number Tandem Repeats; INDEL: Insertion and Deletion; rsid: reference SNPs identification code
