# Supplementary material for: Association between IL1 gene polymorphism and human African trypanosomiasis in populations of sleeping sickness foci of southern Cameroon
Source: PLoS Negl Trop Dis. 2019 Mar 25;13(3):e0007283. doi: 10.1371/journal.pntd.0007283 (PMC6448947; doi:10.1371/journal.pntd.0007283)
Supplement: S3 Table — (DOCX) [file pntd.0007283.s003.docx]

**S3 Table:** Allelic frequencies and values of HWE at each locus for the entire population

| Locus | Allele | Cases (%) | | Controls (%) | OR (95%CI) | HWE |
| --- | --- | --- | --- | --- | --- | --- |
| **VNTR** | ***IL4RN*: rs79071878** | | | | | 0.3739 |
|  | *1R | | 68(46.43) | 259(50.62) | - |  |
|  | **2R | | 74(53.57) | 239(49.38) | 1.147 (0.77-1.70) |  |
|  | ***HP*** | | | | | 0.0965 |
|  | *Hp1 | | 71(46.64) | 281(53.44) | - |  |
|  | **Hp2 | | 71(53.36) | 219(46.56) | 1.254 (0.84-1.87) |  |
|  | ***IL1RN*: rs2234663** | | | | |  |
|  | *1A | | 132(87.04) | 457(94.23) | **-** | **-** |
|  | **4A | | 5(11.11) | 30(5.77) | 0.640 (0.24-1.73) | 1 |
|  | **3A | | 7(1.85) | 9(1.25) | 2.761 (1.01-7.56) | 1 |
| **SNP** | ***IL1A*: rs1800794** | | | | | 0.0002 |
|  | *C | | 80(71.82) | 403(80.50) | - |  |
|  | *T | | 64(28.18) | 89(19.50) | 2.331 (1.50-3.62) |  |
|  | ***IL6*: rs1554606** | | | | | 0.6489 |
|  | *T | | 107(74.11) | 395(77.99) | - |  |
|  | **G | | 39(25.89) | 101(22.01) | 0.950 (0.59-1.53) |  |
|  | ***HPR*: rs1697370** | | | | | 0.1322 |
|  | *T | | 102 | 380 | - |  |
|  | **C | | 44 | 116 | 0.917 (0.56-1.49) |  |
| **INDEL** | ***HLAG*: rs371194629** | | | | | 0.0749 |
|  | *Ins | | 71(48.21) | 250(50.62) | - |  |
|  | **Del | | 75(51.79) | 248(49.32) | 0.764 (0.51-1.15) |  |

OR odds ratio; X²: Chi-square probability value; CI: Confidence Interval at 95%; HWE-P Hardy-Weinberg equilibrium p value for unaffected individuals; **: minor allele; *: major allele; SNP: Single Nucleotide Polymorphism; VNTR: Variable Number Tandem Repeats; INDEL: Insertion and Deletion; *HP*: Haptoglobin; *HPR*: Haptoglobin related protein; IL: Interleukins.
