# Supplementary material for: Association between IL1 gene polymorphism and human African trypanosomiasis in populations of sleeping sickness foci of southern Cameroon
Source: PLoS Negl Trop Dis. 2019 Mar 25;13(3):e0007283. doi: 10.1371/journal.pntd.0007283 (PMC6448947; doi:10.1371/journal.pntd.0007283)
Supplement: S4 Table — (DOCX) [file pntd.0007283.s004.docx]

**S4 Table: Variations of HWE values according to loci and ethno-linguistic groups and subgroups**

| Ethno | Eth. Pools | Eth. Grp | Gene | rsid | Case | Control | HWE |
| --- | --- | --- | --- | --- | --- | --- | --- |
| Bantu | Bantu | | *IL1A* | rs1800794 | 61 | 150 | 0.8951 |
|  |  |  | *IL1RN* | rs2234663 |  |  | 1 |
|  |  |  | *IL4RN* | rs79071878 |  |  | 1 |
|  |  |  | *HLA-G* | rs371194629 |  |  | 1 |
|  |  |  | *IL6* | rs1554606 |  |  | 0.9944 |
|  |  |  | *HPR* | rs1697370 |  |  | 0.2023 |
|  |  |  | *HP* | - |  |  | 0.0623 |
| Semi-Bantu | Semi-Bantu | | *IL1A* | rs1800794 | 07 | 78 | 0.7823 |
|  |  |  | *IL1RN* | rs2234663 |  |  | 1 |
|  |  |  | *IL4RN* | rs79071878 |  |  | 0.9117 |
|  |  |  | *HLA-G* | rs371194629 |  |  | 0.8128 |
|  |  |  | *IL6* | rs1554606 |  |  | 1 |
|  |  |  | *HPR* | rs1697370 |  |  | 0.4162 |
|  |  |  | *HP* | - |  |  | 0.4771 |
| Baka | **Baka** | | *IL1A* | rs1800794 | 05 | 16 | 1 |
|  |  |  | *IL1RN* | rs2234663 |  |  | 1 |
|  |  |  | *IL4RN* | rs79071878 |  |  | 0.911 |
|  |  |  | *HLA-G* | rs371194629 |  |  | 1 |
|  |  |  | *IL6* | rs1554606 |  |  | 1 |
|  |  |  | *HPR* | rs1697370 |  |  | 1 |
|  |  |  | *HP* | - |  |  | 1 |
| Sudano-Sao | **Sudano-Sao** | | *IL1A* | rs1800794 | 00 | 07 | 1 |
|  |  |  | *IL1RN* | rs2234663 |  |  | 1 |
|  |  |  | *IL4RN* | rs79071878 |  |  | 1 |
|  |  |  | *HLA-G* | rs371194629 |  |  | 1 |
|  |  |  | *IL6* | rs1554606 |  |  | 1 |
|  |  |  | *HPR* | rs1697370 |  |  | 1 |
|  |  |  | *HP* | - |  |  | 1 |
| Bantu | **Wovea** | **Douala** | *IL1A* | rs1800794 | 01 | 10 | 1 |
|  |  |  | *IL1RN* | rs2234663 |  |  | 1 |
|  |  |  | *IL4RN* | rs79071878 |  |  | 1 |
|  |  |  | *HLA-G* | rs371194629 |  |  | 0.1998 |
|  |  |  | *IL6* | rs1554606 |  |  | 0.4798 |
|  |  |  | *HPR* | rs1697370 |  |  | 1 |
|  |  |  | *HP* | - |  |  | 1 |
|  |  | **Bassa** | *IL1A* | rs1800794 | 12 | 28 | 1 |
|  |  |  | *IL1RN* | rs2234663 |  |  | 1 |
|  |  |  | *IL4RN* | rs79071878 |  |  | 0.7167 |
|  |  |  | *HLA-G* | rs371194629 |  |  | 1 |
|  |  |  | *IL6* | rs1554606 |  |  | 0.4655 |
|  |  |  | *HPR* | rs1697370 |  |  | 0.2698 |
|  |  |  | *HP* | - |  |  | 0.4097 |
| Bantu | **Beti-Fang** | **Bulu** | *IL1A* | rs1800794 | 00 | 06 | 1 |
|  |  |  | *IL1RN* | rs2234663 |  |  | 1 |
|  |  |  | *IL4RN* | rs79071878 |  |  | 1 |
|  |  |  | *HLA-G* | rs371194629 |  |  | 1 |
|  |  |  | *IL6* | rs1554606 |  |  | 1 |
|  |  |  | *HPR* | rs1697370 |  |  | 1 |
|  |  |  | *HP* | - |  |  | 1 |
|  |  | **Eton** | *IL1A* | rs1800794 | 09 | 13 | 0.0899 |
|  |  |  | *IL1RN* | rs2234663 |  |  | 1 |
|  |  |  | *IL4RN* | rs79071878 |  |  | 0.9539 |
|  |  |  | *HLA-G* | rs371194629 |  |  | 1 |
|  |  |  | *IL6* | rs1554606 |  |  | 1 |
|  |  |  | *HPR* | rs1697370 |  |  | 1 |
|  |  |  | *HP* | - |  |  | 1 |
|  |  | **Fan** | *IL1A* | rs1800794 | 01 | 12 | 1 |
|  |  |  | *IL1RN* | rs2234663 |  |  | 1 |
|  |  |  | *IL4RN* | rs79071878 |  |  | 0.5929 |
|  |  |  | *HLA-G* | rs371194629 |  |  | 1 |
|  |  |  | *IL6* | rs1554606 |  |  | 0.5293 |
|  |  |  | *HPR* | rs1697370 |  |  | 1 |
|  |  |  | *HP* | - |  |  | 1 |
|  |  | **Iyassa** | *IL1A* | rs1800794 | 08 | 23 | 0.6527 |
|  |  |  | *IL1RN* | rs2234663 |  |  | 1 |
|  |  |  | *IL4RN* | rs79071878 |  |  | 1 |
|  |  |  | *HLA-G* | rs371194629 |  |  | 0.8616 |
|  |  |  | *IL6* | rs1554606 |  |  | 0.6232 |
|  |  |  | *HPR* | rs1697370 |  |  | 0.2712 |
|  |  |  | *HP* | - |  |  | 0.2961 |
|  |  | **Kwasse** | *IL1A* | rs1800794 | 06 | 08 | 1 |
|  |  |  | *IL1RN* | rs2234663 |  |  | 1 |
|  |  |  | *IL4RN* | rs79071878 |  |  | 0.5939 |
|  |  |  | *HLA-G* | rs371194629 |  |  | 0.4406 |
|  |  |  | *IL6* | rs1554606 |  |  | 1 |
|  |  |  | *HPR* | rs1697370 |  |  | 1 |
|  |  |  | *HP* | rs10492814 |  |  | 0.4406 |
|  |  | **Maabi** | *IL1A* | rs1800794 | 04 | 09 | 0.4932 |
|  |  |  | *IL1RN* | rs2234663 |  |  | 1 |
|  |  |  | *IL4RN* | rs79071878 |  |  | 0.4932 |
|  |  |  | *HLA-G* | rs371194629 |  |  | 0.5393 |
|  |  |  | *IL6* | rs1554606 |  |  | 1 |
|  |  |  | *HPR* | rs1697370 |  |  | 1 |
|  |  |  | *HP* | - |  |  | 1 |
|  |  | **Mvae** | *IL1A* | rs1800794 | 06 | 09 | 0.1674 |
|  |  |  | *IL1RN* | rs2234663 |  |  | 1 |
|  |  |  | *IL4RN* | rs79071878 |  |  | 1 |
|  |  |  | *HLA-G* | rs371194629 |  |  | 1 |
|  |  |  | *IL6* | rs1554606 |  |  | 1 |
|  |  |  | *HPR* | rs1697370 |  |  | 1 |
|  |  |  | *HP* | - |  |  | 1 |
|  |  | **Ngoumba** | *IL1A* | rs1800794 | 14 | 32 | 0.00004 |
|  |  |  | *IL1RN* | rs2234663 |  |  | 0.0001 |
|  |  |  | *IL4RN* | rs79071878 |  |  | 0.0064 |
|  |  |  | *HLA-G* | rs371194629 |  |  | 0.0025 |
|  |  |  | *IL6* | rs1554606 |  |  | 1 |
|  |  |  | *HPR* | rs1697370 |  |  | 0.0646 |
|  |  |  | *HP* | - |  |  | 0.0725 |
| Semi Bantu | Semi Bantu | **Mundani** | *IL1A* | rs1800794 | 03 | 50 | 1 |
|  |  |  | *IL1RN* | rs2234663 |  |  | 0.5784 |
|  |  |  | *IL4RN* | rs79071878 |  |  | 0.1433 |
|  |  |  | *HLA-G* | rs371194629 |  |  | 1 |
|  |  |  | *IL6* | rs1554606 |  |  | 0.7246 |
|  |  |  | *HPR* | rs1697370 |  |  | 0.1983 |
|  |  |  | *HP* | - |  |  | 1 |
|  |  | **Bamilike** | *IL1A* | rs1800794 | 04 | 12 | 0.886 |
|  |  |  | *IL1RN* | rs2234663 |  |  | 1 |
|  |  |  | *IL4RN* | rs79071878 |  |  | 1 |
|  |  |  | *HLA-G* | rs371194629 |  |  | 1 |
|  |  |  | *IL6* | rs1554606 |  |  | 1 |
|  |  |  | *HPR* | rs1697370 |  |  | 1 |
|  |  |  | *HP* | - |  |  | 1 |
|  |  | **Banyangi** | *IL1A* | rs1800794 | 00 | 12 | 0.2782 |
|  |  |  | *IL1RN* | rs2234663 |  |  | 1 |
|  |  |  | *IL4RN* | rs79071878 |  |  | 0.5542 |
|  |  |  | *HLA-G* | rs371194629 |  |  | 1 |
|  |  |  | *IL6* | rs1554606 |  |  | 1 |
|  |  |  | *HPR* | rs1697370 |  |  | 0.4386 |
|  |  |  | *HP* | - |  |  | 0.0659 |
|  |  | **Wimbum** | *IL1A* | rs1800794 | 00 | 02 | 1 |
|  |  |  | *IL1RN* | rs2234663 |  |  | 1 |
|  |  |  | *IL4RN* | rs79071878 |  |  | 1 |
|  |  |  | *HLA-G* | rs371194629 |  |  | 1 |
|  |  |  | *IL6* | rs1554606 |  |  | 1 |
|  |  |  | *HPR* | rs1697370 |  |  | 1 |
|  |  |  | *HP* | - |  |  | 1 |
|  |  | **Modelle** | *IL1A* | rs1800794 | 00 | 01 | 1 |
|  |  |  | *IL1RN* | rs2234663 |  |  | 1 |
|  |  |  | *IL4RN* | rs79071878 |  |  | 1 |
|  |  |  | *HLA-G* | rs371194629 |  |  | 1 |
|  |  |  | *IL6* | rs1554606 |  |  | 1 |
|  |  |  | *HPR* | rs1697370 |  |  | 1 |
|  |  |  | *HP* | - |  |  | 1 |
|  |  | **Tika** | *IL1A* | rs1800794 | 00 | 01 | 1 |
|  |  |  | *IL1RN* | rs2234663 |  |  | 1 |
|  |  |  | *IL4RN* | rs79071878 |  |  | 1 |
|  |  |  | *HLA-G* | rs371194629 |  |  | 1 |
|  |  |  | *IL6* | rs1554606 |  |  | 1 |
|  |  |  | *HPR* | rs1697370 |  |  | 1 |
|  |  |  | *HP* | - |  |  | 1 |
| Sudano-Sao | | **Foulani** | *IL1A* | rs1800794 | 00 | 01 | 1 |
|  |  |  | *IL1RN* | rs2234663 |  |  | 1 |
|  |  |  | *IL4RN* | rs79071878 |  |  | 1 |
|  |  |  | *HLA-G* | rs371194629 |  |  | 1 |
|  |  |  | *IL6* | rs1554606 |  |  | 1 |
|  |  |  | *HPR* | rs1697370 |  |  | 1 |
|  |  |  | *HP* | - |  |  | 1 |
|  |  | **Moudan** | *IL1A* | rs1800794 | 00 | 05 | 1 |
|  |  |  | *IL1RN* | rs2234663 |  |  | 1 |
|  |  |  | *IL4RN* | rs79071878 |  |  | 1 |
|  |  |  | *HLA-G* | rs371194629 |  |  | 1 |
|  |  |  | *IL6* | rs1554606 |  |  | 1 |
|  |  |  | *HPR* | rs1697370 |  |  | 1 |
|  |  |  | *HP* | - |  |  | 0.3651 |

M.Eth.G: Major ethnic groups; Eth. Pools: pooled Ethno-linguistic subgroups; Eth. Grp: Ethno-linguistic subgroups; HWE-P: Hardy-Weinberg equilibrium p value for unaffected individuals; rsid: reference SNP identification code.

The Bantu contained the Beti-Fang which include the Bulu, Eton, Fan, Iyassa, Kwasse, Maabi, Mvae and Ngoumba ethno-linguistic subgroups. The Wovea is formed by the Douala and Bassa ethno-linguistic subgroups. The Semi-Bantu contained the Mundani, Bamilike, Banyangi, Wimbum, Tika, and Modelle ethno-linguistic subgroups. The Sudano-Sao is formed by the Foulani and Moudan ethno-linguistic subgroups.
