# Supplementary material for: Association between IL1 gene polymorphism and human African trypanosomiasis in populations of sleeping sickness foci of southern Cameroon
Source: PLoS Negl Trop Dis. 2019 Mar 25;13(3):e0007283. doi: 10.1371/journal.pntd.0007283 (PMC6448947; doi:10.1371/journal.pntd.0007283)
Supplement: S5 Table — (DOCX) [file pntd.0007283.s005.docx]

**S5 Table: Fisher analysis results within three major ethnic groups and 10 ethno-linguistic subgroups clusters.**

| Ethno | Gene | rsid | BP | Allele | F_A | F_U | P-value | OR | L95 | U95 | BONF | HWE |
| --- | --- | --- | --- | --- | --- | --- | --- | --- | --- | --- | --- | --- |
| Baka | *IL1A* | rs1800794 | 113543273 | *T  **C | 1 | 0.4 | 0.09173 | NA | NA | NA | 0.6421 | 1 |
|  | *IL1RN* | rs2234663 | 113888106 | *4A  *3A  **1A | 0  0 | 0  0.04545 | 0.5  0.5417 | NA  0 | NA  0 | NA  nan | 1 | 1  1 |
|  | *IL4RN* | rs79071878 | 132680584 | *2R  **1R | 0 | 0.5 | 0.3471 | 0 | 0 | nan | 1 | 0.6167 |
|  | *HLA-G* | rs371194629 | 29830805 | *Del  **Ins | 0.5 | 0.3667 | 0.7581 | 1.727 | 0.09798 | 30.45 | 1 | 0.5778 |
|  | *IL6* | rs1554606 | 154426970 | *G  **T | 0 | 0.2143 | 0.6828 | 0 | 0 | nan | 1 | 0.4899 |
|  | *HPR* | rs1697370 | 35339932 | *C  **T | 0.5 | 0.4333 | 0.746 | 1.308 | 0.07457 | 22.93 | 1 | 0.6148 |
|  | *HP* | - | - | *HP2  **HP1 | 1 | 0.4667 | 0.2419 | NA | NA | NA | 1 | 0.6035 |
| Bamilike | *IL1A* | rs1800794 | 113543273 | *T  **C | 0.5 | 0.3333 | 0.3185 | 2 | 0.3938 | 10.16 | 1 | 1 |
|  | *IL1RN* | rs2234663 | 113888106 | *4A  *3A  **1A | 0.125  0.125 | 0.0417  0 | 0.25  0.125 | 3.286  0 | 0.1811  nan | 59.6  nan | 1  1 | 1  1 |
|  | *IL4RN* | rs79071878 | 132680584 | *2R  **1R | 0.25 | 0.5417 | 0.1675 | 0.2821 | 0.04707 | 1.69 | 1 | 0.5939 |
|  | *HLA-G* | rs371194629 | 29830805 | *Del  **Ins | 0.5 | 0.3333 | 0.3185 | 2 | 0.3938 | 10.16 | 1 | 1 |
|  | *IL6* | rs1554606 | 154426970 | *G  **T | 0.125 | 0.2083 | 0.8124 | 0.5429 | 0.0536 | 5.498 | 1 | 1 |
|  | *HPR* | rs1697370 | 35339932 | *C  **T | 0.375 | 0.3636 | 0.8361 | 1.05 | 0.1968 | 5.602 | 1 | 0.5377 |
|  | *HP* | - | - | *HP2  **HP1 | 0.375 | 0.5 | 0.5573 | 0.6 | 0.1164 | 3.093 | 1 | 1 |
| Bassa | *IL1A* | rs1800794 | 113543273 | *T  **C | 0.4583 | 0.2037 | **0.02129** | 3.308 | 1.168 | 9.366 | 0.149 | 1 |
|  | *IL1RN* | rs2234663 | 113888106 | *4A  *3A  **1A | 0  0 | 0  0.0357 | 0.5  0.7563 | NA  NA | NA  NA | NA  NA | 1  1 | 1  1 |
|  | *IL4RN* | rs79071878 | 132680584 | *2R  **1R | 0.2917 | 0.4464 | 0.1786 | 0.5106 | 0.183 | 1.424 | 1 | 0.7167 |
|  | *HLA-G* | rs371194629 | 29830805 | *Del  **Ins | 0.4167 | 0.5357 | 0.3434 | 0.619 | 0.2355 | 1.627 | 1 | 1 |
|  | *IL6* | rs1554606 | 154426970 | *G  **T | 0.25 | 0.4643 | 0.06577 | 0.3846 | 0.1329 | 1.113 | 0.4604 | 0.4655 |
|  | *HPR* | rs1697370 | 35339932 | *C  **T | 0.2083 | 0.2679 | 0.6813 | 0.7193 | 0.228 | 2.269 | 1 | 0.0068 |
|  | *HP* | - | - | *HP2  **HP1 | 0.6667 | 0.3393 | **0.0103** | 3.895 | 1.414 | 10.73 | 0.0721 | 0.4249 |
| Douala | *IL1A* | rs1800794 | 113543273 | *T  **C | 0 | 0.1111 | 0.5974 | 0 | 0 | nan | 1 | 1 |
|  | *IL1RN* | rs2234663 | 113888106 | *4A  *3A  **1A | 0  0 | 0  0.0556 | 0.5  0.55 | NA  0 | NA  nan | NA  nan | 1  1 | 1  1 |
|  | *IL4RN* | rs79071878 | 132680584 | *2R  **1R | 0.5 | 0.4444 | 0.7395 | 1.25 | 0.06718 | 23.26 | 1 | 0.5393 |
|  | *HLA-G* | rs371194629 | 29830805 | *Del  **Ins | 0 | 0.4444 | 0.3211 | 0 | 0 | nan | 1 | 0.0555 |
|  | *IL6* | rs1554606 | 154426970 | *G  **T | 0 | 0.2778 | 0.7237 | 0 | 0 | nan | 1 | 1 |
|  | *HPR* | rs1697370 | 35339932 | *C  **T | 0.5 | 0 | 0.05 | NA | NA | NA | 0.35 | 1 |
|  | *HP* | - | - | *HP2  **HP1 | 0.5 | 0.5 | 0.7368 | 1 | 0.05384 | 18.57 | 1 | 1 |
| Eton | *IL1A* | rs1800794 | 113543273 | *T  **C | 0.5556 | 0.3077 | 0.09395 | 2.812 | 0.8073 | 9.799 | 0.6577 | 0.0334 |
|  | *IL1RN* | rs2234663 | 113888106 | *4A  *3A  **1A | 0  0.0556 | 0.0385  0.0417 | 0.7045  0.7491 | 0  1.353 | 0  0.0789 | Nan  23.2 | 1  1 | 1  1 |
|  | *IL4RN* | rs79071878 | 132680584 | *2R  **1R | 0.5 | 0.4615 | 0.8833 | 1.167 | 0.3502 | 3.887 | 1 | 1 |
|  | *HLA-G* | rs371194629 | 29830805 | *Del  **Ins | 0.5 | 0.5 | 0.8779 | 1 | 0.2946 | 3.395 | 1 | 1 |
|  | *IL6* | rs1554606 | 154426970 | *G  **T | 0.3889 | 0.4615 | 0.6509 | 0.7424 | 0.2188 | 2.519 | 1 | 1 |
|  | *HPR* | rs1697370 | 35339932 | *C  **T | 0.1667 | 0.3846 | 0.1399 | 0.32 | 0.07358 | 1.392 | 0.9794 | 1 |
|  | *HP* | - | - | *HP2  **HP1 | 0.5625 | 0.4231 | 0.4405 | 1.753 | 0.4986 | 6.165 | 1 | 1 |
| Fan | *IL1A* | rs1800794 | 113543273 | *T  **C | 1 | 0.4167 | 0.1015 | NA | NA | NA | 0.7108 | 0.2893 |
|  | *IL1RN* | rs2234663 | 113888106 | *4A  *3A  **1A | 0  0 | 0.04545  0 | 0.5417  0.5 | 0  0 | 0  0 | nan  nan | 1  1 | 1  1 |
|  | *IL4RN* | rs79071878 | 132680584 | *2R  **1R | 1 | 0.375 | 0.08462 | NA | NA | NA | 0.5923 | 0.5929 |
|  | *HLA-G* | rs371194629 | 29830805 | *Del  **Ins | 1 | 0.375 | 0.08462 | NA | NA | NA | 0.5923 | 1 |
|  | *IL6* | rs1554606 | 154426970 | *G  **T | 1 | 0.25 | **0.04308** | NA | NA | NA | 0.3015 | 0.5293 |
|  | *HPR* | rs1697370 | 35339932 | *C  **T | 0 | 0.375 | 0.32 | 0 | 0 | nan | 1 | 0.2053 |
|  | *HP* | - | - | *HP2  **HP1 | 0.5 | 0.4583 | 0.7415 | 1.182 | 0.06596 | 21.17 | 1 | 1 |
| Iyassa | *IL1A* | rs1800794 | 113543273 | *T  **C | 0.6875 | 0.3864 | **0.03205** | 3.494 | 1.033 | 11.82 | 0.2244 | 0.6527 |
|  | *IL1RN* | rs2234663 | 113888106 | *4A  *3A  **1A | 0  0 | 0.04348  0.04348 | 0.7263  0.7263 | 0  0 | 0  0 | nan  nan | 1  1 | 1  1 |
|  | *IL4RN* | rs79071878 | 132680584 | *2R  **1R | 0.4375 | 0.4348 | 0.8853 | 1.011 | 0.3211 | 3.183 | 1 | 0.6741 |
|  | *HLA-G* | rs371194629 | 29830805 | *Del  **Ins | 0.5 | 0.413 | 0.657 | 1.421 | 0.4277 | 4.722 | 1 | 0.6701 |
|  | *IL6* | rs1554606 | 154426970 | *G  **T | 0.625 | 0.3043 | **0.0265** | 3.81 | 1.158 | 12.54 | 0.1855 | 0.3651 |
|  | *HPR* | rs1697370 | 35339932 | *C  **T | 0.25 | 0.2174 | 0.6155 | 1.2 | 0.3171 | 4.541 | 1 | 0.2507 |
|  | *HP* | - | - | *HP2  **HP1 | 0.4375 | 0.4783 | 0.8899 | 0.8485 | 0.2701 | 2.665 | 1 | 0.1048 |
| Kwasse | *IL1A* | rs1800794 | 113543273 | *T  **C | 0.5 | 0.1429 | 0.06261 | 6 | 0.9187 | 39.18 | 0.483 | 1 |
|  | *IL1RN* | rs2234663 | 113888106 | *4A  *3A  **1A | 0.1  0 | 0  0 | 0.1923  0.5 | NA  NA | NA  NA | NA  NA | 1  1 | 1  1 |
|  | *IL4RN* | rs79071878 | 132680584 | *2R  **1R | 0.5 | 0.4375 | 0.8588 | 1.286 | 0.2863 | 5.774 | 1 | 0.5301 |
|  | *HLA-G* | rs371194629 | 29830805 | *Del  **Ins | 0.4167 | 0.375 | 0.8523 | 1.19 | 0.2577 | 5.499 | 1 | 0.4406 |
|  | *IL6* | rs1554606 | 154426970 | *G  **T | 0.25 | 0.3125 | 0.8454 | 0.7333 | 0.1366 | 3.938 | 1 | 1 |
|  | *HPR* | rs1697370 | 35339932 | *C  **T | 0.4167 | 0.4375 | 0.8511 | 0.9184 | 0.202 | 4.175 | 1 | 0.5301 |
|  | *HP* | rs10492814 | 72123886 | *HP2  **HP1 | 0.3333 | 0.375 | 0.849 | 0.8333 | 0.1734 | 4.006 | 1 | 0.4406 |
| Maabi | *IL1A* | rs1800794 | 113543273 | *T  **C | 0.75 | 0.3889 | 0.1159 | 4.714 | 0.734 | 30.28 | 0.8112 | 0.4932 |
|  | *IL1RN* | rs2234663 | 113888106 | *4A  *3A  **1A | 0  0.375 | 0.05556  0 | 0.6538  0.0108 | 0  0 | 0  0 | nan  nan | 1  0.0754 | 1  1 |
|  | *IL4RN* | rs79071878 | 132680584 | *2R  **1R | 0.375 | 0.3889 | 0.8322 | 0.9429 | 0.1694 | 5.248 | 1 | 0.4932 |
|  | *HLA-G* | rs371194629 | 29830805 | *Del  **Ins | 0.375 | 0.5556 | 0.4372 | 0.48 | 0.0871 | 2.645 | 1 | 0.5393 |
|  | *IL6* | rs1554606 | 154426970 | *G  **T | 0.5 | 0.4444 | 0.8414 | 1.25 | 0.2356 | 6.633 | 1 | 0.1707 |
|  | *HPR* | rs1697370 | 35339932 | *C  **T | 0.25 | 0 | **0.04308** | NA | NA | NA | 0.305 | 1 |
|  | *HP* | - | - | *HP2  **HP1 | 0.375 | 0.5 | 0.5419 | 0.6 | 0.1092 | 3.296 | 1 | 1 |
| Mvae | *IL1A* | rs1800794 | 113543273 | *T  **C | 0.5 | 0.3125 | 0.347 | 2.2 | 0.4676 | 10.35 | 1 | 0.0769 |
|  | *IL1RN* | rs2234663 | 113888106 | *4A  *3A  **1A | 0.08333  0.1667 | 0  0 | 0.2143  0.0873 | NA  NA | NA  NA | NA  NA | 1  0.6111 | 1  1 |
|  | *IL4RN* | rs79071878 | 132680584 | *2R  **1R | 0.5833 | 0.4375 | 0.4777 | 1.8 | 0.396 | 8.182 | 1 | 5.301 |
|  | *HLA-G* | rs371194629 | 29830805 | *Del  **Ins | 0.25 | 0.5 | 0.1871 | 0.3333 | 0.06509 | 1.707 | 1 | 1 |
|  | *IL6* | rs1554606 | 154426970 | *G  **T | 0.4167 | 0.25 | 0.3275 | 2.143 | 0.4276 | 10.74 | 1 | 0.3846 |
|  | *HPR* | rs1697370 | 35339932 | *C  **T | 0.1667 | 0.125 | 0.8066 | 1.4 | 0.1678 | 11.68 | 1 | 1 |
|  | *HP* | - | - | *HP2  **HP1 | 0.4167 | 0.4375 | 0.8511 | 0.9184 | 0.202 | 4.175 | 1 | 1 |
| Mundani | *IL1A* | rs1800794 | 113543273 | *T  **C | 0.5 | 0.2872 | 0.2676 | 2.481 | 0.4711 | 13.07 | 1 | 1 |
|  | *IL1RN* | rs2234663 | 113888106 | *4A  *3A  **1A | 0  0 | 0.1596  0 | 0.4038  0.5 | 0  0 | 0  0 | nan  nan | 1  1 | 0.5784  1 |
|  | *IL4RN* | rs79071878 | 132680584 | *2R  **1R | 0.5 | 0.4583 | 0.842 | 1.182 | 0.227 | 6.153 | 1 | 0.1433 |
|  | *HLA-G* | rs371194629 | 29830805 | *Del  **Ins | 0.5 | 0.4688 | 0.8407 | 1.133 | 0.2177 | 5.9 | 1 | 1 |
|  | *IL6* | rs1554606 | 154426970 | *G  **T | 0.3333 | 0.3438 | 0.8307 | 0.9545 | 0.1661 | 5.487 | 1 | 0.0510 |
|  | *HPR* | rs1697370 | 35339932 | *C  **T | 0.5 | 0.3723 | 0.5372 | 1.686 | 0.3224 | 8.813 | 1 | 0.0056 |
|  | *HP* | - | - | *HP2  **HP1 | 0.6667 | 0.4792 | 0.3186 | 2.174 | 0.3801 | 12.43 | 1 | 0.7721 |

P-value: Nominal P unadjusted asymptotic probability value; *: minor allele; **: major allele: OR: odds ratio; BONF: Bonferroni adjusted asymptotic p value; F_A & F_U frequency of the minor allele in cases and controls respectively; Ethno: Ethno-linguistic groups; BP base-pair location; HWE-P Hardy-Weinberg equilibrium p value for unaffected individuals; [L95-U95] = Lower and Upper limit of 95% confidence interval of odds ratio; rsid: Reference SNP identification code
